# Three-dimensional morphological analysis of the thoracic pedicle and related radiographic factors in adolescent idiopathic scoliosis

**DOI:** 10.1186/s12891-022-05799-4

**Published:** 2022-09-07

**Authors:** Tatsuya Sato, Hidetoshi Nojiri, Takatoshi Okuda, Kei Miyagawa, Nozomu Kobayashi, Ryosuke Takahashi, Arihisa Shimura, Shota Tamagawa, Yukoh Ohara, Takeshi Hara, Muneaki Ishijima

**Affiliations:** 1 Department of Orthopedic Surgery, Juntendo University School of Medicine, 2-1-1 Hongo, Bunkyoku, Tokyo, Japan; 2 Department of Neurosurgery, Juntendo University, Tokyo, Japan

**Keywords:** Thoracic spine, Adolescent idiopathic scoliosis, Three-dimensional computed tomography, Pedicle morphometry, Pedicle screw

## Abstract

**Background:**

This study aimed to investigate the laterality of the pedicle morphology at the apical vertebra (AV) level and identify the radiographic factors associated with the laterality ratio of the pedicle morphology at the AV level in patients with adolescent idiopathic scoliosis (AIS).

**Methods:**

Overall, 684 pedicles in 57 AIS patients aged 10–20 years, who underwent preoperative computed tomography (CT) and had Lenke type 1 or 2 with right convex main thoracic curves (MTC), were evaluated. Pedicle diameters of the MTC were assessed. We defined and compared the region containing two vertebrae adjacent to the AV (APEX±1) and the region containing two vertebrae adjacent to the neutral vertebra. We analyzed the pedicle diameter and laterality ratio of APEX±1 and performed multiple linear regression analysis to identify the radiographic factors associated with the laterality of the pedicle diameter.

**Results:**

On the concave side of APEX±1, the pedicles of 15 patients (26.3%) did not accept a 4-mm-diameter pedicle screw (PS), even with 25% cortical bone width expansion. Laterality ratio differences in the pedicle diameters of the cortical bone width in APEX±1 were large in patients with more proximal AV level (*p* < 0.001) and smaller apical vertebral rotation (AVR) (*p* = 0.029).

**Conclusions:**

Preoperative planning to accurately select and insert the PS in AIS should be based on the anatomical limitations in APEX±1, AV level, and AVR degree. In APEX±1, the correlation between AVR and the laterality ratio of the pedicle diameter may be useful for pathoetiological interpretation of the AIS deformity.

**Supplementary Information:**

The online version contains supplementary material available at 10.1186/s12891-022-05799-4.

## Introduction

Pedicle screw (PS) fixation at multiple levels is commonly used worldwide in spinal surgery for three-dimensional (3D) correction of adolescent idiopathic scoliosis (AIS) [1, 2]. However, a misplaced PS can result in neurological, vascular, and visceral complications [3]. The pedicle morphology in AIS is narrower than that in a normal spine and varies highly among the different curve types [4]. Therefore, high accuracy in PS placement is required [5–7], and preoperative planning of PS placement is essential [8]. In particular, PS insertion in the apical vertebra (AV) region on the concave side provides a positive correction effect; therefore, it is preferred whenever possible [9]. Right-left side-to-side differences in the pedicle dimensions at and near the apex in AIS patients are greater than those in healthy individuals [10], and PS insertion cannot be performed because the pedicle width is small, particularly on the concave side [11].

Recently, the use of computed tomography (CT) images has become common and important for the preoperative assessment of PS insertion [12]. However, in previous reports, the pedicle diameter was often evaluated using a single-slice CT image reconstructed based on the “pedicle axis”, which was subjectively set by the radiologist [13–15]. In AIS with a complex 3D deformation, the pedicle axis differs significantly in each vertebral body. Therefore, the error in pedicle diameter measurement is large and depends on how the radiologist has fixed the slice position in the pedicle. Previous studies have investigated the use of cadavers in obtaining accurate pedicle measurements from 3D images [16–18]. However, there is no report of accurate evaluation of the pedicle diameter by reconstructing the image of each vertebral body and the plane orthogonal to the left and right pedicle axes using a CT-based 3D image.

This study aimed to investigate the laterality of the thoracic pedicle morphology in the AV region of the main thoracic curve (MTC) in AIS patients using a large series of 3D-CT reconstructed images. The study also aimed to identify the radiographical factors associated with the laterality ratio of the pedicle morphology in the AV region of the MTC. We defined and used the term “laterality ratio” as a relative, rather than absolute, value. The laterality ratio was used as a measurement of the laterality of the pedicle diameter within an individual patient with high accuracy. We defined the “laterality ratio” as “the pedicle width index: the ratio of the left and right pedicle diameters of the same vertebral body,” as previously described [19].

## Materials and methods

### Patient identification

Japanese patients with AIS aged 10–20 years, who undergone preoperative CT examination between January 2012 and December 2021 and had single or double thoracic curves of Lenke curve type 1 or 2, were included. Children with other spinal pathologies or atypical left convex thoracic curves were excluded. In this study, the right pedicle was always the convex pedicle, and the left pedicle was always the concave pedicle. This study was approved by the medical ethics committee of a university hospital, and informed consent was obtained from the patients and their parents.

### Radiographical measurements

All the patients underwent a routine upright posteroanterior, lateral, and bending radiographic examinations before surgery. The degree of the MTC was assessed using the Cobb angle protocol. Additionally, the Risser grade [20] was measured. The lumbar modifiers were classified into types A, B, and C based on the Lenke classification [21]. The AV level was measured at the MTC. Apical vertebral translation (AVT) was measured as the distance between the central sacral vertical line and the center of the AV of the MTC (both right and left; all as positive values). Thoracic kyphosis (TK) was measured as the sagittal Cobb between the T5 and T12 vertebrae. CT imaging (Aquilion ONE; Canon Medical Systems, Japan) was performed preoperatively from the thoracic to lumbar vertebrae using a slice thickness of 0.5 mm, a gantry rotation time of 0.5 s, atube voltage of 120 kVp, and a pitch factor of 0.8. No additional CT scanning was performed for the purpose of this study. Apical vertebral rotation (AVR) was measured from the preoperative axial CT at the MTC using the methods described by Aaro and Dahlborn [22].

### Morphologic evaluation with 3D reconstruction software

**Fig. 1:**
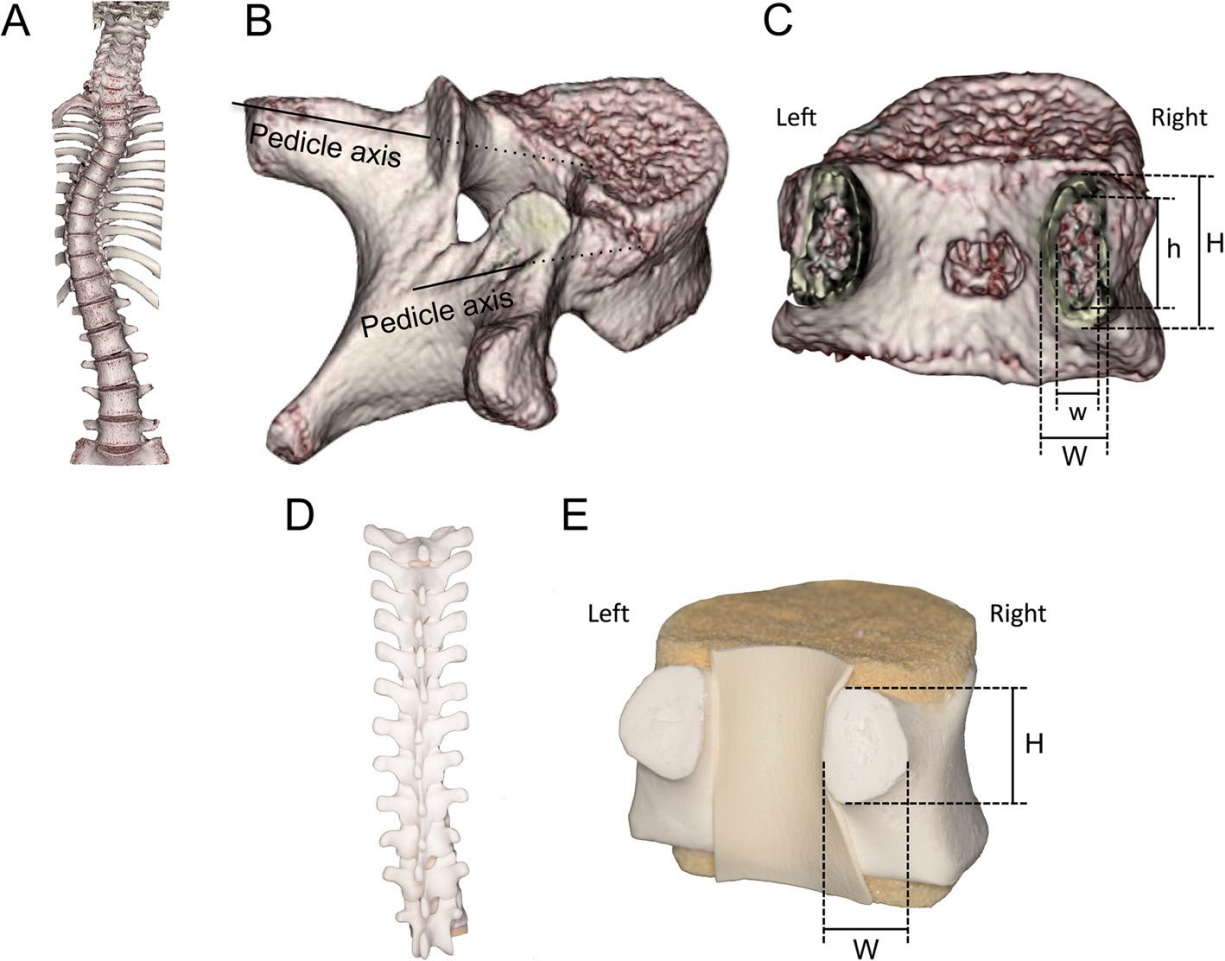
Morphologic imaging evaluation using 3D reconstruction software (Synapse Vincent® version 6.1; FUJIFILM Medical Co., Ltd., Tokyo, Japan). **a** The software automatically generated a three-dimensional (3D) reproduction of the whole spine. **b** The observer manually isolated the vertebral bodies and cuts out the pedicles in a plane perpendicular to the pedicle axis through the isthmus. **c** The pedicle height and width in both the cortical and cancellous bones in APEX±1 and NV ± 1 were measured using the cross section of the pedicles of the 3D spinal model. APEX±1, the region of the apical vertebra and its two adjacent vertebrae; NV ± 1, the region of the cranial NV and its two adjacent vertebrae; H, cortical pedicle height; h, cancellous pedicle height; W, cortical pedicle width; w, cancellous pedicle width. **d** Overview of the thoracic model bone. **e** T10 thoracic vertebra image was cut in a plane containing isthmus, orthogonal to the anatomical pedicle axis. H, pedicle height; W, pedicle width. The laterality ratio of the pedicle diameter of the cortical bone width was defined as Lt.W/Rt.W, and the laterality ratio of the pedicle diameter of the cancellous bone width was defined as Lt.W/Rt.W. The laterality ratio of the pedicle diameter of the cortical bone height was defined as Lt.H/Rt.H, and the laterality ratio of the pedicle diameter of the cancellous bone width was defined as Lt.h/Rt.h

A picture archiving and communication system was used to transfer the CT data to a workstation, and measurements were made using a 3D reconstruction software program (Synapse Vincent® version 6.1; FUJIFILM Medical Co., Ltd., Tokyo, Japan). The pedicle diameter was measured using 3D-CT images constructed with Synapse Vincent® in the following four-step procedure: (1) registration of the CT data and creation of the virtual 3D spinal model, (2) isolation of the vertebral bodies, (3) cutting out pedicles in a plane perpendicular to the pedicle axis through the isthmus, and (4) measurement of the cross section of the pedicles (Fig. 1A, B, and C). The pedicle axes were set freehand by multiple examiners on a 3D reconstructed image of the trajectory by which the surgeon inserted an anatomical PS. We used a thoracic bone model to evaluate the observer bias and reliability of the measured values of the pedicle cross section obtained from the pedicle axes (Fig. 1D and E).

To evaluate the laterality of the pedicle diameter near the AV, we grouped the pedicles into two regions based on the AV and proximal neutral vertebra (NV). The AV region (APEX±1) was defined by the AV and its two adjacent vertebrae. The control region (NV ± 1) included the NV and its two adjacent vertebrae. We set the NV to upper NV instead of lower NV, because the lower NV is often located in the lumbar spine, which is structurally different from the thoracic spine. Furthermore, the absolute value of the pedicle diameter is significantly larger in the lumbar spine than in the thoracic spine [23], and it has a greater effect on the left/right pedicle diameter ratio. Moreover, the lower NV is strongly influenced by the lumbar modifier. The observed pedicle morphometric parameters were shown in Fig. 1 and were represented by the mean values of the pedicle height and width in both cortical and cancellous bones in APEX±1 and NV ± 1 (Fig. 1A, B, and C). Similarly, for 12 thoracic spine model bones, bilateral pedicle cross sections were cut out, and the height and width were measured both empirically and by Synapse Vincent® (Fig. 1D and E). We termed the left/right pedicle height of the cortical bone as Lt.H/Rt.H, the left/right pedicle width of the cortical bone as Lt.W/Rt.W, the left/right pedicle height of the cancellous bone as Lt.h/Rt.h, and the left/right pedicle width of the cancellous bone as Lt.w/Rt.w.

### Statistical analysis

Statistical analyses were performed using IBM SPSS Statistics, version 21 (IBM Japan, Tokyo). A dependent t-test was used to compare the left and right pedicle diameters of APEX±1 and NV ± 1. Pearson’s correlation coefficient (r) and multiple linear regression analysis were used to analyze the relationship between the left and right pedicle diameter differences with patient characteristics or radiographic features. Finally, intra- and interobserver variabilities were obtained as intraclass correlation coefficients. For intra- and interobserver reliability, the observers analyzed a random subset of 10 CT images separately (observer 1, twice; observer 2, once). To verify the reliability of Synapse Vincent®, the intra- and interobserver reliabilities of the two measurements of each of the 12 thoracic bone models (24 pedicles) and two measurements of 3D-CT images constructed with Synapse Vincent® were also analyzed. A *p*-value < 0.05 was considered statistically significant.

## Results

A total of 57 patients were recruited for this study. There were 49 women (86.0%) and 8 men (14.0%), with a mean age of 16.3 ± 2.4 years. The mean height was 160.0 ± 6.7 cm and the mean weight was 49.2 ± 7.9 kg. The mean body mass index was 19.3 ± 2.4 kg/m^2^. Lenke types 1 and 2 were observed in 25 (43.9%) and 32 (56.1%) patients respectively. Lumbar modifiers A and B-C were present in 45 (78.9%) and 12 (21.1%) patients, respectively. Risser grades 0–3 and 4–5 were observed in 7 (12.3%) and 50 (87.7%) patients, respectively. The following variables were measured for the MTC: the mean Cobb angle was 58.5 ± 8.9°; the level of the upper NV varied between T4 and T8; the level of the AV varied between T6 and T12; the length of the curves was 7.7 ± 1.0 vertebrae; the mean AVT was 57.1 ± 17.2 mm; the mean AVR was 19.4 ± 6.7°; and the mean TK was 15.5 ± 6.7° (Table 1).

**Table 1:** Patient and radiographical characteristics

Variables		Total (*N* = 57)
***Patient Characteristics***		
Age, in years		16.2 ± 2.4 (12–20)
Sex, N (%)	Male	8 (14.0%)
	Female	49 (86.0%)
Hight, in cm		160 ± 6.7 (145–176)
Weight, in kg		49.2 ± 7.9 (36–72)
BMI, in %		19.3 ± 2.4 (14.9–25.4)
***Radiographical Characteristics***		
Lenke Type, N (%)	1	25 (43.9%)
	2	32 (56.1%)
Lenke Modifier, N (%)	A	45 (78.9%)
	B or C	12 (21.1%)
Risser Grade, N (%)	0–3	7 (12.3%)
	4 or 5	50 (87.7%)
Level of Upper NV	T4	4 (7.0%)
	T5	11 (19.3%)
	T6	29 (50.9%)
	T7	11 (19.3%)
	T8	2 (3.5%)
Level of AV	T6	1 (1.8%)
	T7	3 (5.3%)
	T8	4 (7.0%)
	T9	28 (49.1%)
	T10	14 (24.6%)
	T11	6 (10.5%)
	T12	1 (1.8%)
Cobb Angle of MTC, in degrees		58.5 ± 8.9 (43–81)
Number of MTC Vertebra		7.7 ± 1.0 (6–10)
AVT, in mm		57.1 ± 17.2 (10.2–94.9)
AVR, in degrees		19.4 ± 6.7 (9.0–42.0)
TK, in degrees		15.5 ± 6.7 (6–36)

*AV* apical vertebra; *AVR* apical vertebral rotation; *AVT* apical vertebral translation; *BMI* body mass index; *MTC* main thoracic curve; *NV* neutral vertebra; *TK* thoracic kyphosis

For the 57 observed AIS patients with primary right thoracic (Lenke types 1–2) curves, a total of 342 thoracic vertebrae were identified in the 3D-CT images constructed with Synapse Vincent®, forming a database of 684 corresponding (left and right) pedicles.

### Reliability

The intraclass correlation coefficients of the pedicle measurements using 3D-CT images constructed by Synapse Vincent® for the verification of intra- and interobserver reliabilities were 0.965 (95% confidence interval, 0.939–0.980) and 0.982 (0.969–0.990), respectively. Considering the reliability using thoracic bone models of Synapse Vincent®, intraclass correlation coefficients for intra- and interobserver reliabilities were 0.996 (95% confidence interval, 0.990–0.998) and 0.999 (0.996–0.999), respectively.

### Right versus left pedicle morphometry in APEX±1 and NV ± 1

The observed pedicle morphometric parameters were shown in Table 2 and were represented by the mean values of the pedicle height and width in the cortical and cancellous bones in APEX±1 and NV ± 1. The difference between the left and right pedicle diameters were not significant in the cortical and cancellous widths of NV ± 1. However, this difference was significant in APEX±1. In APEX±1, a Rt. W < 4 mm was observed in 6 (10.5%) patients, a Rt. W < 3.2 mm that could not hold a 4-mm-diameter screw (even with a 25% pedicle expansion) was observed in 1 (1.7%) patient, a Lt. W < 4 mm was observed in 41 (71.9%) patients, and a Lt.W < 3.2 mm was observed in 15 (26.3%) patients. Furthermore, in APEX±1, there was significant laterality of more than 1.0 mm in all of the heights and widths of the cortical and cancellous bones of pedicles. In particular, for the width of the cortical bone, differences between the left and right pedicle diameters were > 1.0 mm in 41 patients (71.9%, 1.1–2.9 mm) and ≤ 1.0 mm in 16 patients (28.1%, 0–1.0 mm). The right and left pedicle diameter ratios in APEX±1 and NV ± 1 were shown in Table 3. Lt.w/Rt.w showed the most significant left-right difference among the right and left diameter ratios in APEX±1.

**Table 2:** Differences in right and left pedicle diameters

Variables			Rt. Pedicles	Lt. Pedicles	*p*-values
APEX ±1	Cortical Bone	H (mm)	12.4 ± 1.5 (9.1–16.3)	10.8 ± 1.5 (7.3–15.6)	< 0.001*
		W (mm)	4.9 ± 0.8 (2.9–6.2)	3.6 ± 0.9 (0.9–5.7)	< 0.001*
	Cancellous Bone	h (mm)	7.5 ± 1.4 (4.9–11.4)	5.9 ± 1.5 (1.9–10.1)	< 0.001*
		w (mm)	2.6 ± 0.7 (1.2–4.0)	1.7 ± 0.7 (0.2–3.9)	< 0.001*
NV ±1	Cortical Bone	H (mm)	10.8 ± 1.5 (7.3–15.6)	9.1 ± 1.0 (6.9–11.8)	< 0.001*
		W (mm)	3.6 ± 0.9 (0.9–5.6)	3.6 ± 0.7 (2.4–5.7)	0.929
	Cancellous Bone	h (mm)	5.9 ± 1.5 (1.9–10.1)	4.9 ± 1.2 (2.4–8.1)	< 0.001*
		w (mm)	1.7 ± 0.7 (0.2–3.9)	1.8 ± 0.7 (0.7–4.1)	0.903

*Statistically significant. APEX±1, the region of the AV and its two adjacent vertebrae; H, cortical pedicle height; h, cancellous pedicle height; NV ± 1, the region of the NV and its two adjacent vertebrae; W, cortical pedicle width; w, cancellous pedicle width

**Table 3:** Right and left pedicle diameter ratios in APEX±1 and NV ± 1

Variables		APEX ±1	NV ±1	*p*-values
Cortical Bone	Lt. H / Rt. H	0.86 ± 0.52 (0.75–0.97)	0.97 ± 0.06 (0.82–1.10)	< 0.001*
	Lt. W / Rt. W	0.73 ± 0.13 (0.28–1.00)	1.08 ± 0.17 (0.77–1.54)	< 0.001*
Cancellous Bone	Lt. h / Rt. h	0.79 ± 0.13 (0.36–1.11)	1.06 ± 0.22 (0.58–1.65)	< 0.001*
	Lt. w / Rt. w	0.67 ± 0.24 (0.11–1.23)	1.38 ± 0.48 (0.53–2.73)	< 0.001*

*Statistically significant. *APEX±1* the region of the apical vertebra and its two adjacent vertebrae; *Lt.H/Rt.H* left/right pedicle height of the cortical bone; *Lt.h/Rt.h* left/right pedicle height of the cancellous bone; *Lt.W/Rt.W* left/right pedicle width of the cortical bone; *Lt.w/Rt.w* left/right pedicle width of the cancellous bone; *NV ± 1* the region of the NV and its two adjacent vertebrae

### Correlation between right-left pedicle morphological differences with patient characteristics or radiographical features

**Fig. 2:**
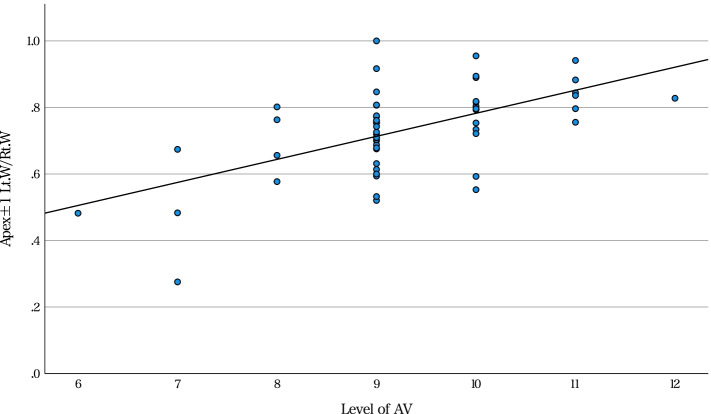
Correlation of Lt.W/Rt.W in APEX±1 with the AV level Lt.W/Rt.W in APEX±1 positively correlated with the level of the AV (*r* = 0.550, *p* < 0.001). AV, apical vertebra; Lt.W, left cortical pedicle width; Rt.W, right cortical pedicle width

**Fig. 3:**
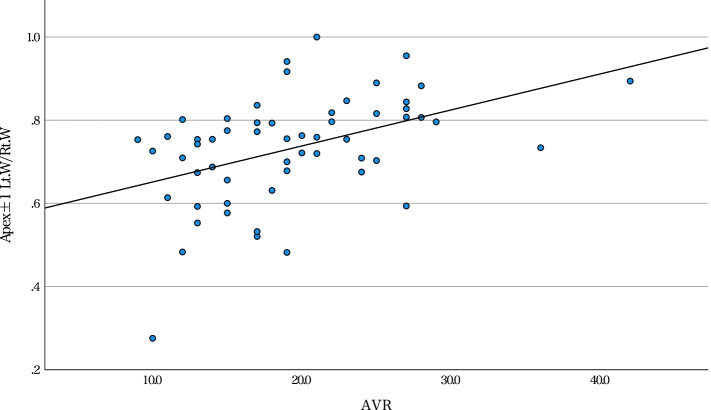
Correlation of Lt.W/Rt.W in APEX±1 with the AVR. Lt.W/Rt.W in APEX±1 positively correlated with the AVR (*r* = 0.483, *p* < 0.001). APEX±1, the region of the apical vertebra and its two adjacent vertebrae; AVR, apical vertebral rotation; Lt.W/Rt.W, the left/right pedicle width of cortical bone

Univariate analysis suggested that right-left pedicle morphological differences tended to have a significantly positive correlation with several radiographical characteristics (Table 4). Furthermore, Lt.W/Rt.W in APEX±1 had a significantly moderate correlation with the level of the AV and AVR (Figs. 2 and 3). Among the patients, the distribution of AV was concentrated in T9 and T10. Therefore, we further performed a subgroup analysis of patients with AV T9 or T10. In that subgroup, Lt.W/Rt.W correlated with the level of the AV and AVR (Table 5). Multiple linear regression analysis showed that Lt.W/Rt.W in APEX±1 could be predicted by the level of the AV and AVR (Table 6). The coefficient of determination (R^2^) was 0.396.

**Table 4:** Correlation coefficients between patient characteristics and radiographical features with and right and left pedicle diameter ratios

Variables			Age	Height	Weight	BMI	Level of AV	Cobb Angle of MTC	Number of MTC	AVT	AVR	TK
APEX ±1	Cortical Bone	Lt. H / Rt. H	0.090	0.093	−0.063	−0.061	0.236	0.131	0.195	0.040	0.158	0.047
		Lt. W / Rt. W	−0.026	− 0.022	0.053	0.097	0.550^*^	0.100	0.163	0.296^*^	0.483^*^	0.023
	Cancellous Bone	Lt. h / Rt. h	0.099	−0.084	0.008	0.116	0.484^*^	−0.111	0.293^*^	−0.015	0.031	−0.075
		Lt. w / Rt. w	−0.060	−0.005	− 0.022	−0.005	0.658^*^	0.113	0.277^*^	0.364^*^	0.365^*^	−0.071
NV ±1	Cortical Bone	Lt. H / Rt. H	0.166	−0.191	0.008	0.074	0.390^*^	−0.067	0.138	0.203	0.209	−0.163
		Lt. W / Rt. W	−0.061	−0.205	− 0.218	−0.147	0.487^*^	0.18	0.267^*^	0.384^*^	0.326^*^	−0.253
	Cancellous Bone	Lt. h / Rt. h	0.018	−0.097	−0.175	− 0.129	0.375^*^	0.165	0.062	0.323^*^	0.247	−0.172
		Lt. w / Rt. w	−0.088	−0.152	− 0.103	0.002	0.631^*^	0.304^*^	0.298^*^	0.530^*^	0.390^*^	−0.117

*Statistically significant. *AV* apical vertebra; *AVR* apical vertebral rotation; *AVT* apical vertebral translation; *BMI* body mass index; *Lt.H/Rt.H* left/right pedicle height of the cortical bone; *Lt.h/Rt.h* left/right pedicle height of the cancellous bone; *Lt.W/Rt.W* left/right pedicle width of the cortical bone; *Lt.w/Rt.w* left/right pedicle width of the cancellous bone; *MTC* main thoracic curve; *TK* thoracic kyphosis

**Table 5:** Correlation coefficients between patient characteristics and radiographical features with right and left pedicle diameter ratios in the subgroup with AV of T9 or T10

Variables			Age	Height	Weight	BMI	Level of AV	Cobb Angle of MTC	Number of MTC	AVT	AVR	TK
APEX ±1	Cortical Bone	Lt. H / Rt. H	0.074	0.074	−0.188	−0.145	0.179	0.202	0.141	−0.005	0.032	−0.024
		Lt. W / Rt. W	−0.041	0.057	0.071	0.119	0.308^*^	0.190	0.042	0.205	0.389^*^	−0.047
	Cancellous Bone	Lt. h / Rt. h	0.132	−0.052	−0.066	0.079	0.171	−0.062	0.16	−0.194	−0.193	− 0.235
		Lt. w / Rt. w	−0.081	0.112	0.003	−0.007	0.392^*^	0.178	0.162	0.238	0.178	−0.183
NV ±1	Cortical Bone	Lt. H / Rt. H	0.246	−0.119	0.122	0.191	0.400^*^	−0.128	0.105	0.150	0.138	−0.195
		Lt. W / Rt. W	−0.089	−0.176	− 0.227	−0.133	0.313^*^	0.300*	0.163	0.366^*^	0.227	−0.405^*^
	Cancellous Bone	Lt. h / Rt. h	0.009	−0.073	−0.186	− 0.140	0.025	0.187	−0.106	0.218	0.087	−0.280
		Lt. w / Rt. w	−0.077	−0.179	− 0.142	−0.010	0.354^*^	0.394^*^	0.121	0.445^*^	0.209	−0.298

*Statistically significant. *AV* apical vertebra; *AVR* apical vertebral rotation; *AVT* apical vertebral translation; *BMI* body mass index; *Lt.H/Rt.H* left/right pedicle height of the cortical bone; *Lt.h/Rt.h* left/right pedicle height of the cancellous bone; *Lt.W/Rt.W* left/right pedicle width of the cortical bone; *Lt.w/Rt.w* left/right pedicle width of the cancellous bone; *MTC* main thoracic curve; *TK* thoracic kyphosis

**Table 6:** Multiple linear regression analysis of predictors of Lt.W/Rt.W

Variables	Unstandardized Coefficients		Standardized Coefficients	95% CI for B		*p*-values
	B	Standerd Error	β	Lower	Upper	
Level of AV	0.058	0.014	0.485	0.38	0.85	< 0.001
AVR	0.005	0.002	0.256	0.001	0.010	0.029

The coefficient of determination (R^2^) was 0.396. *AV* apical vertebra; *AVR* apical vertebral rotation; *CI* confidence interval; *Lt.W/Rt.W* left/right pedicle width of the cortical bone

## Discussion

Accurate measurement of the pedicle diameter in AIS surgery is important to prevent complications from PS malposition [3, 23] and to obtain a PS fixation with the target pedicle occupancy rate of > 80% [24, 25]. In particular, PS insertion in APEX±1 has been reported to have a positive effect on curve correction [9], and the measurement of the pedicle diameter in APEX±1 based on the AV level is highly critical for surgical planning. While the AV level differs by the curve types, there has been no pedicle morphological analysis based on the AV position in each patient. Furthermore, when evaluating pedicle diameters on CT images, if the pedicle morphometry is not performed on the plane that accurately determines the pedicle axis, the risk of overestimating the true pedicle diameter increases [26]. Therefore, in this study, we measured pedicle diameter on 3D-CT images, and we measured the isthmus of the pedicle orthogonal to the pedicle axis.

Pedicle diameter differences were significantly larger by a maximum of approximately 1.6 mm on the right side than on the left side in APEX±1, and the laterality ratio of the pedicle diameter tended to be large at Lt.w/Rt.w and small at Lt.H/Rt.H. However, the Lt.w/Rt.w of cancellous bone in NV ± 1 was 1.38, and we should not have discussed cancellous bone widths in APEX±1 using NV ± 1 as the control region. The reason may be that the pedicles of the upper thoracic vertebrae of AIS are often atypical and do not have cancellous bone [12], and the cancellous bone widths at NV ± 1 vary extensively. The predictors of Lt.W/Rt.W were the level of the AV and AVR. Interestingly, this new finding indicated that more proximal AV level or smaller AVR was associated with smaller Lt.W/Rt.W (i.e., larger laterality ratio of the pedicle diameter of the cortical bone width in APEX±1). To examine the laterality of the pedicle diameter in each patient, we referred to a previous study [19] for the definition of the “laterality ratio”. Furthermore, although there were no correlations between height, weight, and gender with pedicle width in this study, the thoracic pedicle diameter is generally influenced by height, weight, or gender in individuals without AIS [27, 28]. These reports also showed the reason a left-right difference comparison was not conducted using the absolute value of the pedicle diameter. Moreover, there is no consensus on whether the left-right ratio or the left-right absolute value difference should be used to compare the left-right morphology of the pedicle diameter. The closer the level of AV is to T4 or 5, the smaller the pedicle width is [4]. Nevertheless, we found that the closer the level of AV was to T4 or 5, the greater the value of Rt.W-Lt.W was (Supplementary Table 1A, 1B, and 1C). Therefore, we considered that the use of Lt.W/Rt.W to discuss the laterality of the pedicle diameter in this study was appropriate.

The most important determinant of the PS diameter is the width of the cortical bone [27]. Since a pedicle allows 15–25% enlargement [23, 28, 29], for a PS insertion of 4.0 mm, the outer diameter should be at least > 3.2 (4.0/1.25) mm. Our data suggested that 15 (26.3%) patients, presenting an APEX±1 pedicle on the concave side, would sustain a fracture if a screw for the thoracic spine was inserted. For patients in whom the PS could not be inserted at the concave side of APEX±1, we might consider using hook or sublayer wiring [30, 31]. Convex side correction with PS inserted into the convex side might also be considered a useful correction method [27]. Moreover, the in-out-in insertion techniques could be considered if insertion of the screw into the pedicle is determined to be challenging owing to the small width of the pedicle. However, while the in-out-in insertion technique minimizes the risk of nerve damage, sympathetic trunk damage might occur [16]. This technique was also associated with reduced pull-out strength compared to PS placed using an anatomical orbit [32]; thus, inserting a pedicle screw within the permissible range of pedicle enlargement is preferred.

In previous evaluations of the mean value for each vertebral body level, the difference between the left and right pedicle diameters of the middle and lower thoracic vertebrae was within 1.0 mm in cortical pedicle height, cancellous pedicle height, cortical pedicle width, and cancellous pedicle width [11, 23]. However, a new finding from this study showed that the left and right pedicle diameters significantly differed by > 1.0 mm. In particular, 41 (71.9%) patients had pedicle width differences > 1.0 mm, which are important for planning PS insertion. The reason might be that the recruited participants had only AIS types 1 and 2. Additionally, the left and right pedicle diameters in APEX±1 was compared based on the AV, and the measurement was performed on 3D-CT reconstructed images.

Two notably major findings from this study could be useful for spine surgeons. First, predicting pedicle morphology from the AV level is possible without preoperative CT. Second, at APEX±1, laterality of the cortical pedicle width on the opposite side of the AVR direction might exist (e.g., the pedicle width on the opposite side of the AVR direction is relatively large).

This study did not suggest the need for preoperative CT for the prevention of PS misplacement in AIS patients. Currently, both intra-operative navigation and, to an even greater extent, CT-based preoperative planning, are extremely useful tools. However, CT-based preoperative planning did not significantly reduce PS misplacement [33], and their use could be limited to selected cases. Therefore, understanding differences in pedicle morphology is considered even more important for the surgeons to prevent PS misplacement in patients who do not undergo preoperative CT.

Based on the results of this study, we concluded that greater vertebral body rotation in APEX±1 was associated with smaller laterality ratio (i.e., the larger Lt.W/Rt.W). In AIS patients, it was previously reported that larger curve magnitude of the coronal plane was associated with a larger increase in pedicle dysplasia at the apex and concave side of the curve [12, 34]. However, in this study, the Cobb angle of MTC was not a related factor of Lt.W/Rt.W in APEX±1. In other words, our results suggested that the vertebral rotation, an axial evaluation, affected Lt.W/Rt.W more significantly than the curve magnitude, a coronal plane evaluation. This phenomenon could be related to a pathoetiological mechanism, by which the AVR and spinous processes are deformed to compensate for each other in the axial plane [35]. Another possible explanation was that the pedicle diameters were evaluated not as the absolute value but as the left-right ratio of a particular vertebral body in the multiple linear regression analysis of this study. Therefore, our results might differ from those of previous reports [12, 34].

This study had several limitations. First, computerized measurements might not capture the entire natural biological variability of the pedicle shape as well as every pathological shape. However, the advantage of computerized measurements is that they allow the extraction of additional pedicle morphometric parameters and thus, can reliably support spine surgeons in selecting proper screw sizes as well as inserting trajectories during preoperative planning for PS placement procedures. Second, PS diameters through the isthmus might differ by several millimeters, depending on the selection of the insertion points and differences in the pedicle axis and PS insertion axis, because the surgeon did not always insert the PS in the same trajectory as the pedicle axis when considering rotation correction and the PS placement sequence.

## Conclusions

In AIS with Lenke type 1 or 2, 26.3% of the pedicles in APEX±1 on the concave side were too small for a 4-mm-diameter screw even with an expansion. The right and left pedicle diameter ratio in APEX±1 for the height or width of the cortical or cancellous bone ranged from 0.7 to 0.9. There was a notable difference in the pedicle diameter ratio between the left and right pedicle widths of the cancellous bone (Lt.W/Rt.W). Furthermore, the pedicle diameter ratio between the left and right widths of the cortical bone was larger in patients with more proximal AV and smaller AVR. Although preoperative planning of each AIS patient remains important for surgeons, this study may help plan the selection of available instrumentation from information, other than the preoperative CT, that could predict the laterality of the pedicle morphology at APEX±1 from the preoperative curve type.

## Supplementary Information

**Additional file 1.**

## Data Availability

The datasets used and analyzed during the present study are available from the corresponding author on reasonable request. **Software application code:** 22000BZX00238000.

## Abbreviations

3D: Three-dimensional
AIS: Adolescent idiopathic scoliosis
AV: Apical vertebra
AVR: Apical vertebral rotation
AVT: Apical vertebral translation
CI: Confidence interval
CT: Computed tomography
MTC: Main thoracic curve
NV: Neutral vertebra
PS: Pedicle screw
TK: Thoracic kyphosis
