# Supplementary material for: Three-dimensional morphological analysis of the thoracic pedicle and related radiographic factors in adolescent idiopathic scoliosis
Source: BMC Musculoskelet Disord. 2022 Sep 7;23:847. doi: 10.1186/s12891-022-05799-4 (PMC9450434; doi:10.1186/s12891-022-05799-4)
Supplement: Supplementary file 1 — Additional file 1. [file 12891_2022_5799_MOESM1_ESM.docx]

| Level of AV | Rt.W (mm) | Lt.W (mm) | Lt.W/Rt.W | Rt.W - Lt.W (mm) |
| --- | --- | --- | --- | --- |
| T6 | 5.7 | 2.7 | 0.48 | 3.0 |
| T7 | 3.7 ±1.0 (3.0–4.8) | 1.9 ±1.2 (0.9–3.2) | 0.47 ±0.20 (0.28–0.67) | 1.8 ±0.5 (1.5–2.3) |
| T8 | 5.0 ±0.6 (4.2–5.4) | 3.5 ±0.4 (3.1–4.0) | 0.70 ±0.10 (0.58–0.80) | 1.6 ±0.6 (0.8–2.3) |
| T9 | 4.8 ±0.8 (3.2–6.2) | 3.5 ±0.7 (1.7–4.9) | 0.72 ±0.10 (0.52–1.0) | 1.3 ±0.5 (0.0–2.7) |
| T10 | 4.8 ±0.6 (3.6–5.9) | 3.8 ±0.9 (2.1–5.7) | 0.78 ±0.11 (0.55–0.96) | 1.1 ±0.5 (0.3–2.4) |
| T11 | 5.4 ±0.7 (4.3–6.1) | 4.5 ±0.7 (3.5–5.3) | 0.84 ±0.07 (0.75–0.94) | 0.8 ±0.4 (0.3–1.5) |
| T12 | 5.8 | 4.8 | 0.83 | 1.0 |

**Supplement Table 1A.** Absolute value difference and ratio of the left and right widths of each cortical pedicle in APEX ±1 by Level of AV. Absolute value difference and ratio of the left and right of each cortical pedicle widths in APEX ±1 by Level of AV.

AV, apical vertebra; Rt.W, right pedicle width of the cortical bone; Lt.W left pedicle width of the cortical bone; Rt.W -Lt.W, right minus left pedicle width of the cortical bone.

**Supplement Table 1B.** The Correlation Coefficient Between Patient Characteristics, Radiographical Characteristics and Right and Left Pedicle Diameter Difference

| Variables | | | Age | Height | Weight | BMI | Level of AV | Cobb Angle of MTC | Number of MTC | AVT | AVR | TK |
| --- | --- | --- | --- | --- | --- | --- | --- | --- | --- | --- | --- | --- |
| APEX ±1 | Cortical Bone | Rt.H - Lt.H | 0.058 | -0.062 | 0.064 | 0.133 | -0.047 | -0.109 | -0.098 | 0.072 | 0.014 | 0.002 |
|  |  | Rt.W - Lt.W | 0.050 | 0.218 | 0.085 | -0.029 | -0.513* | -0.142 | -0.149 | -0.246 | -0.381* | -0.025 |
|  | Cancellous Bone | Rt.h - Lt.h | 0.076 | 0.039 | -0.103 | -0.145 | -0.361* | 0.184 | -0.259 | 0.133 | 0.132 | 0.056 |
|  |  | Rt.w - Lt.w | 0.161 | 0.200 | 0.148 | 0.060 | -0.570* | -0.095 | -0.231 | -0.226 | -0.227 | 0.071 |
| NV ±1 | Cortical Bone | Rt.H - Lt.H | -0.162 | 0.233 | 0.081 | -0.061 | -0.367* | 0.062 | -0.126 | -0.151 | -0.176 | 0.238 |
|  |  | Rt.W - Lt.W | 0.040 | 0.206 | 0.215 | 0.130 | -0.506* | -0.200 | -0.293* | -0.428* | -0.316* | 0.231 |
|  | Cancellous Bone | Rt.h - Lt.h | 0.008 | 0.198 | 0.203 | 0.131 | -0.419* | -0.230 | -0.044 | -0.308* | -0.258 | 0.254 |
|  |  | Rt.w - Lt.w | 0.090 | 0.174 | 0.143 | 0.071 | -0.550* | -0.224 | -0.282* | -0.437* | -0.346* | 0.176 |

BMI, body mass index; AV, apical vertebra; MTC, main thoracic curve; AVT, apical vertebral translation; AVR, apical vertebral rotation; TK, thoracic kyphosis; APEX±1, the region of the apical vertebra and its two adjacent vertebrae; NV±1, the region of the neutral vertebra and its two adjacent vertebrae; H, cortical pedicle height; h, cancellous pedicle height; W, cortical pedicle width; w, cancellous pedicle width; Rt.H - Lt.H, right minus left pedicle height of the cortical bone; Rt.W - Lt.W, right minus left pedicle width of the cortical bone; Rt.h - Lt.h, right minus left pedicle height of the cancellous bone; Rt.w - Lt.w, right minus left pedicle width of the cancellous bone

**Supplement Table 1C.** The Correlation Coefficient Between Patient Characteristics, Radiographical Characteristics and Right and Left Pedicle Diameter Difference in the Subgroup that AV was T9 or T10

| Variables | | | Age | Height | Weight | BMI | Level of AV | Cobb Angle of MTC | Number of MTC | AVT | AVR | TK |
| --- | --- | --- | --- | --- | --- | --- | --- | --- | --- | --- | --- | --- |
| APEX ±1 | Cortical Bone | Rt.H - Lt.H | 0.093 | -0.011 | 0.211 | 0.275 | -0.059 | -0.154 | -0.097 | 0.070 | 0.082 | 0.043 |
|  |  | Rt.W - Lt.W | 0.055 | 0.049 | 0.062 | 0.035 | -0.252 | -0.248 | 0.009 | -0.211 | -0.359* | 0.074 |
|  | Cancellous Bone | Rt.h - Lt.h | 0.094 | 0.062 | -0.011 | -0.053 | 0.060 | 0.162 | -0.194 | 0.260 | 0.352* | 0.167 |
|  |  | Rt.w - Lt.w | 0.165 | -0.019 | 0.115 | 0.157 | -0.319* | -0.140 | -0.148 | -0.133 | -0.138 | 0.170 |
| NV ±1 | Cortical Bone | Rt.H - Lt.H | -0.251 | 0.140 | -0.057 | -0.161 | -0.377* | 0.145 | -0.097 | -0.081 | -0.092 | 0.274 |
|  |  | Rt.W - Lt.W | 0.084 | 0.142 | 0.131 | 0.068 | -0.330* | -0.282 | -0.179 | -0.403* | -0.245 | 0.386* |
|  | Cancellous Bone | Rt.h - Lt.h | -0.021 | 0.141 | 0.177 | 0.121 | -0.057 | -0.217 | 0.093 | -0.215 | -0.114 | 0.348* |
|  |  | Rt.w - Lt.w | 0.148 | 0.113 | 0.031 | -0.022 | -0.296 | -0.270 | -0.110 | -0.370* | -0.179 | 0.330* |

*Statistically significant.

BMI, body mass index; AV, apical vertebra; MTC, main thoracic curve; AVT, apical vertebral translation; AVR, apical vertebral rotation; TK, thoracic kyphosis; APEX±1, the region of the apical vertebra and its two adjacent vertebrae; NV±1, the region of the neutral vertebra and its two adjacent vertebrae; H, cortical pedicle height; h, cancellous pedicle height; W, cortical pedicle width; w, cancellous pedicle width; Rt.H-Lt.H, right minus left pedicle height of the cortical bone; Rt.W - Lt.W, right minus left pedicle width of the cortical bone; Rt.h - Lt.h, right minus left pedicle height of the cancellous bone; Rt.w - Lt.w, right minus left pedicle width of the cancellous bone
